# Experimental Phantom-Based Security Analysis for Next-Generation Leadless Cardiac Pacemakers

**DOI:** 10.3390/s18124327

**Published:** 2018-12-07

**Authors:** Muhammad Faheem Awan, Sofia Perez-Simbor, Concepcion Garcia-Pardo, Kimmo Kansanen, Narcis Cardona

**Affiliations:** 1 Department of Electronic Systems, Norwegian University of Science and Technology, NTNU, NO-7491 Trondheim, Norway; kimmo.kansanen@ntnu.no; 2 iTEAM, Universitat Politècnica de València, 46022 Valencia, Spain; sopresim@iteam.upv.es (S.P.-S.); cgpardo@iteam.upv.es (C.G.-P.); ncardona@iteam.upv.es (N.C.)

**Keywords:** implanted medical devices, wireless leadless cardiac pacemaker, WBAN, security and privacy, physical-layer security, phantom experiments, channel modeling

## Abstract

With technological advancement, implanted medical devices can treat a wide range of chronic diseases such as cardiac arrhythmia, deafness, diabetes, etc. Cardiac pacemakers are used to maintain normal heart rhythms. The next generation of these pacemakers is expected to be completely wireless, providing new security threats. Thus, it is critical to secure pacemaker transmissions between legitimate nodes from a third party or an eavesdropper. This work estimates the eavesdropping risk and explores the potential of securing transmissions between leadless capsules inside the heart and the subcutaneous implant under the skin against external eavesdroppers by using physical-layer security methods. In this work, we perform phantom experiments to replicate the dielectric properties of the human heart, blood, and fat for channel modeling between in-body-to-in-body devices and from in-body-to-off-body scenario. These scenarios reflect the channel between legitimate nodes and that between a legitimate node and an eavesdropper. In our case, a legitimate node is a leadless cardiac pacemaker implanted in the right ventricle of a human heart transmitting to a legitimate receiver, which is a subcutaneous implant beneath the collar bone under the skin. In addition, a third party outside the body is trying to eavesdrop the communication. The measurements are performed for ultrawide band (UWB) and industrial, scientific, and medical (ISM) frequency bands. By using these channel models, we analyzed the risk of using the concept of outage probability and determine the eavesdropping range in the case of using UWB and ISM frequency bands. Furthermore, the probability of positive secrecy capacity is also determined, along with outage probability of a secrecy rate, which are the fundamental parameters in depicting the physical-layer security methods. Here, we show that path loss follows a log-normal distribution. In addition, for the ISM frequency band, the probability of successful eavesdropping for a data rate of 600 kbps (Electromyogram (EMG)) is about 97.68% at an eavesdropper distance of 1.3 m and approaches 28.13% at an eavesdropper distance of 4.2 m, whereas for UWB frequency band the eavesdropping risk approaches 0.2847% at an eavesdropper distance of 0.22 m. Furthermore, the probability of positive secrecy capacity is about 44.88% at eavesdropper distance of 0.12 m and approaches approximately 97% at an eavesdropper distance of 0.4 m for ISM frequency band, whereas for UWB, the same statistics are 96.84% at 0.12 m and 100% at 0.4 m. Moreover, the outage probability of secrecy capacity is also determined by using a fixed secrecy rate.

## 1. Introduction

Rapid development in personal health systems due to wireless body area networks (WBAN) has resulted in a number of implantable and wearable medical devices. These on-body and in-body wireless medical devices continuously monitor different physiological conditions and provide proper diagnosis and treatment. Notable among these devices are cardiac pacemakers and implanted cardiac defibrillators (ICDs).

Pacemakers are used to treat different types of cardiac arrhythmias. Annually, there are about 0.7 million pacemaker implantations worldwide [1]. A pacemaker senses irregularities between heartbeats and provides proper actuation via electrodes, thus facilitating the proper functioning of human heart. Currently these pacemakers are mostly implanted with wired connection between subcutaneous implants and electrodes in right ventricle and right atrium of the human heart. The next generation of these pacemakers is expected to be wireless between subcutaneous implants and electrodes (EU Horizon 2020 Project WiBEC${}^{\prime \prime }$ Wireless In-Body Environment) [2]. The only currently available leadless pacemaker on the market is Medtronic’s Micra [3], which is an autonomous leadless pacemaker implanted in the right ventricle of a human heart, whereas our project focuses on multi-nodal leadless pacemakers with subcutaneous implant. Our work focuses on the analysis of the eavesdropping risk and secrecy rate between a node implanted in the right ventricle and another node as subcutaneous implanted, but it can be applicable to other scenarios for in-body communications. These scenarios may include communication between nodes within a heart.

The wireless nature of modern implanted medical devices (IMDs) is a significant source of security risks. It makes an IMD more visible and can allow an eavesdropper to listen. Thus, an insecure communication channel makes it easier for an eavesdropper to perform attacks on an implant similar to attacks on other computing devices. Successful eavesdropping may result in the retrieval of patient information (medical and non-medical) or performing attacks such as data forging or altering. In addition, it may enable the modification of the implant configuration without the knowledge of the patient or physician.

The aim of this work is to estimate the channel models for legitimate and eavesdropper links by phantom experiments. Estimated channel models are then used to determine the eavesdropping risk in respective bands along with providing information regarding theoretical secrecy analysis i.e., the availability of the secure channel based on secrecy capacity, which can be directly applied, without any leakage of information to the eavesdropper. We focus on the communication between the leadless cardiac pacemaker (LCP) and subcutaneous implant in frequencies from 1.7–2.5 GHz (ISM band) and 3.1–5.1 GHz (UWB). We develop path-loss models for an in-body-to-in-body (IB2IB) scenario (a legitimate link between the leadless pacemaker in the right ventricle of the human heart and the subcutaneous implant under the skin below the shoulder) and an in-body-to-off-body (IB2OFF) scenario (eavesdropper link between the leadless pacemaker and the eavesdropper outside the body). All results are provided for ultrawide band (UWB) and industrial, scientific, and medical (ISM) frequency bands, and comparison is provided where applicable. Our key contributions in this paper are:

- Single and multilayer phantoms for heart muscle, fat, and blood are developed for respective frequency bands.
- Channel modeling of both legitimate link (IB2IB) and eavesdropper link (IB2OFF).
- Comparison of channel models obtained from measurements performed with different phantoms.
- Probability of successful eavesdropping with respect to eavesdropper distance.
- Secrecy capacity analysis and evaluation of the probability of positive secrecy capacity.
- Design principles or privacy by design where outage probability of a secure rate is determined.

The rest of the paper is organized as follows. Section 2 provides background and problem description, followed by measurement setup in Section 3. Section 4 provides channel models based on measurements. Secrecy analysis including eavesdropping risk and positive secrecy capacity is provided in Section 5 and Section 6. Section 7 discusses the results and Section 8 concludes the work.

## 2. Background & Problem Description

### 2.1. Background

The work of Halperin et al. [4] is considered to be the pioneer work in security analysis of IMDs, followed by different research activities providing security for IMD devices [5]. Most of the research is focused on mitigating the security risks via providing different encryption mechanisms to protect data between a sender and legitimate nodes [6,7,8]. In conventional wireless networks, security is considered to be an independent feature with no or little connection to other tasks of a communication network. State-of-the-art encryption algorithms are developed for such purposes and are implemented and studied via cryptographic algorithms (e.g., RSA, AES, DES etc.) [9,10]. These methods rely on the limited computational power of an eavesdropper and require proper key management servers for implementation, which cannot be the case for tiny IMDs. In addition, the computational complexity is also higher because of data encryption with the key.

An alternative could be to offer secure communication via information theoretic measures or physical-layer security. The concept of information theoretic security was first introduced by Shannon [11], which was further extended by Wyner [12] with introduction to the wiretap channel. The idea behind information theoretic security is to limit the leakage of information to an eavesdropper. A secure communication channel by information theoretic measures can be achieved in two different ways: one is to secure communication without keys, and can be referred to as keyless security, whereas the other is to secure communication with keys. Keyless security can be achieved using appropriate coding schemes with the aid of secrecy capacity [13,14]. Secrecy capacity is the maximum attainable communication rate without leakage of information to an eavesdropper. The second method uses random channel characteristics, e.g., received signal strength (RSS), phase or channel state information (CSI), to generate a key on the physical layer for data encryption. The key-generation methods rely on channel reciprocity. A good deal of research is devoted to key generation using channel reciprocity [15,16,17,18,19].

The focal point of information theoretic security or physical-layer security is using the characteristics of wireless channels. If one can estimate the wireless channels between legitimate nodes and eavesdropper, then the secure rate for communication can be determined. Thus, to provide physical-layer security using the keyless security method, the essential part is to predict the legitimate and eavesdropper channels. The channel characteristics can be achieved by measuring the channel transfer functions of the legitimate and eavesdropper link. In addition, it can help determine the channel capacities, respectively. If the eavesdropper channel’s signal-to-noise ratio (SNR) is lower than that of the legitimate channel, then the difference between the link capacities provides the secrecy capacity for communication. Furthermore, secrecy capacity is the maximum achievable transmission rate keeping eavesdropper uncertainty about the source message to maximum. To determine the secrecy capacity of a system, the knowledge about channels between the legitimate link and the eavesdropper link is required.

Channel characterizations are usually done by software simulations and experimental measurements that include in vivo and phantom experiments. It is difficult to simulate these channels in practice using in vivo experiments because of moral, ethical, and physical integrity reasons. Similarly, software simulations are computationally very costly and requires a good deal of time. A cheap and better alternative is to characterize human body channels via phantom experiments [20]. Phantoms are chemical mixtures that can be used to mimic the electromagnetic behavior of different human body parts provided by Gabriel [21]. From phantom experiments, the amount of data gathered is of considerable size compared to that of in vivo and software simulations, and can lead to better estimation of channels.

In Wireless Body Area Network (WBAN) standard IEEE 802.15.6, Medical Implant Communication System (MICS) frequency band is allocated for implant-to-implant communication that spans 402–405 MHz. Literature is also available on channel modelling in other frequency bands using phantom experiments for in-body nodes [22,23,24,25,26]. In [27], the off-body-to-in-body mathematical model based on software simulations is also provided for the propagation of electromagnetic waves through various tissues/layers by considering reflections from different layers. In our work, we opt for phantom experiments to measure the channels because of less complexity and ease of use, considering the random angles of both the receivers (legitimate receiver and eavesdropper). We focus on ISM and UWB. These frequency bands are under study for implant communications due to prospects such as high data rate and smaller antenna dimensions.

### 2.2. Problem Description

**Figure 1:**
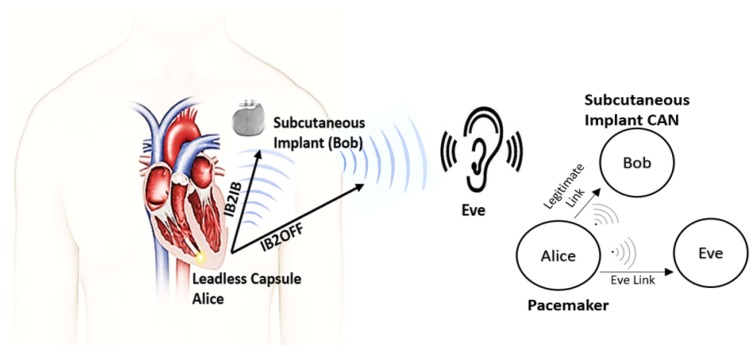
System Model.

The real-world scenario which we want to replicate using phantom experiments is shown in Figure 1. Our system includes a LCP inside the right ventricle of a human heart that communicates with a subcutaneous implant whereas an eavesdropper wants to eavesdrop the communication outside the body. Thus, for security analysis, first we determine the channel models for legitimate and eavesdropper links using phantom experiments in both ISM and UWB frequency bands. We consider a transmission of a sounding signal in the respective bands using vector analyzer (VNA), through different mediums that constitute heart muscle, blood, and fat, emulated as phantoms. Different antennas are used to replicate a leadless pacemaker in right ventricle, a subcutaneous implant, and an eavesdropper. Moreover, the antennas used for ISM and UWB frequency bands are also different. Afterwards these channel models are used to predict the eavesdropping risk and secrecy capacity analysis. In this paper, the link between a pacemaker and the subcutaneous implant is referred to as a legitimate link or in-body-to-in-body (IB2IB) link, whereas the link between pacemaker and eavesdropper is referred as the eavesdropper link or in-body-to-off-body (IB2OFF) link. In addition, we assume that the eavesdropper is of a passive nature and the legitimate nodes are authenticated by some authentication protocol.

## 3. Measurement Setup

**Figure 2:**
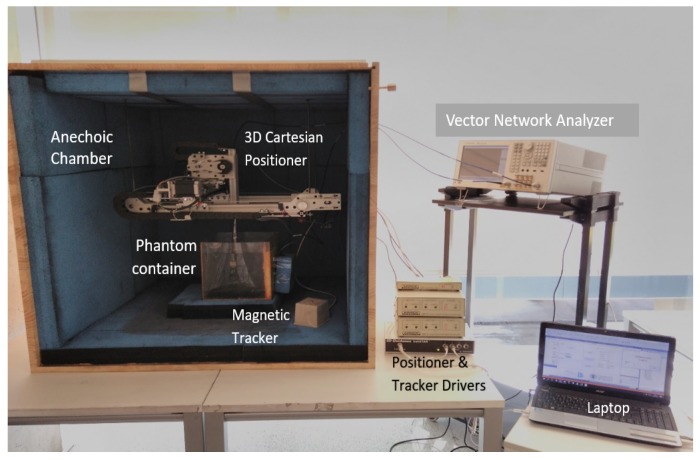
Measurement Setup.

The setup used for measuring the legitimate and eavesdropper channel is shown in Figure 2. It contains an anechoic chamber, a Vector Network Analyzer (VNA), a 3D spatial positioner, a phantom container, and a magnetic tracker. The anechoic chamber is used to reduce the surrounding environmental contributions, the magnetic tracker measures the distance between transmitter and receiver antenna at different measuring points, whereas the positioner is used to precisely move an antenna to different measuring points. The VNA is controlled via a laptop with software that performs initial calibration of components before measurement and configures all the devices. First, it is calibrated with *Rosenberger calibration kit RPC-3.50* to remove the losses due to coaxial cables. Afterwards, it automatically measures the coupling between antennas at the specified grid points. The phantom temperature is maintained at 24 ${}^{\circ }$C because of the variation in permittivity due to temperature change. The phantoms are developed to emulate at room temperature (24 ${}^{\circ }$C), the electromagnetic properties of the human body at 37 ${}^{\circ }$C provided by Gabriel in [21]. More details about the anechoic chamber and measurement setup can be found in [26]. Table 1, shows the set of parameters used for ISM and UWB frequency-band measurements. When everything is in place, before starting automatic measurements across different grid points, the anechoic chamber is closed from the front to keep it completely concealed from outdoor surroundings.

### 3.1. Phantom Composition and Antenna Description

In phantom-based experiments, a container is filled with liquid phantom that mimics the dielectric properties of a required human tissue/body organ. Considering our real-world scenario, the phantoms that depict the dielectric properties of a human heart, fat, and blood are developed. The dielectric properties of a human body vary in frequency, resulting in different compositions of phantoms for different frequency bands. First, the phantom formation for the ISM frequency band is presented along with antennas used. Then, the UWB frequency band will follow.

#### 3.1.1. ISM Band

**Figure 3:**
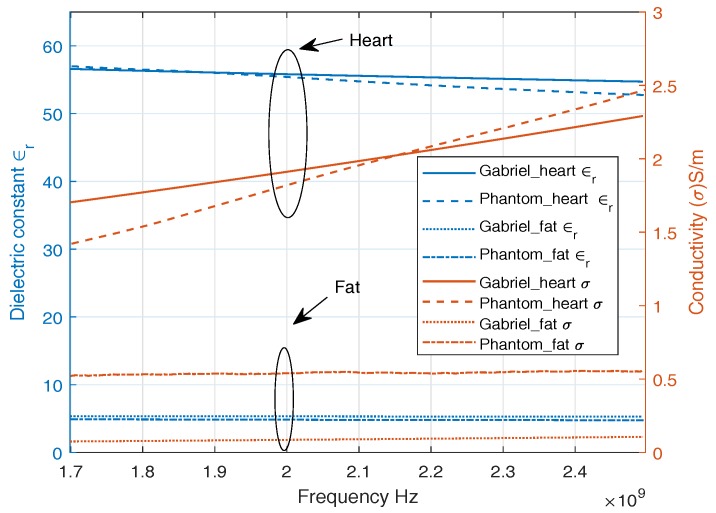
Dielectric Properties of ISM Phantom.

ISM band is a common band to be employed in medical environments due to being license free. We develop phantoms with dielectric properties of a human heart, fat, and blood. Figure 3 shows the dielectric properties of a replicated phantom with its counterpart reported in [21], widely used in literature. A good approximation of dielectric properties of heart muscle, fat, and blood is observed around 2 GHz. A single-layer phantom consists of only heart muscle which is composed of 39.2% sugar with the remainder water [28]. Multilayer phantom includes heart muscle, blood, and fat. The fat phantom is composed of 86% of oil in water where 1% of TX-100 was used as surfactant [29]. The blood phantom is composed of 40% acetonitrile and 1.25% NaCl [30] in water.

**Figure 4:**
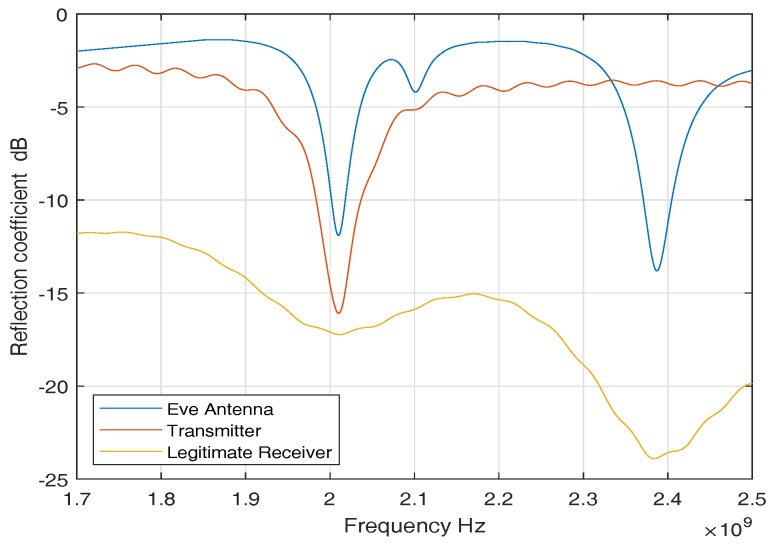
Reflection coefficients for Legitimate and Eavesdropper antennas (ISM).

**Figure 5:**
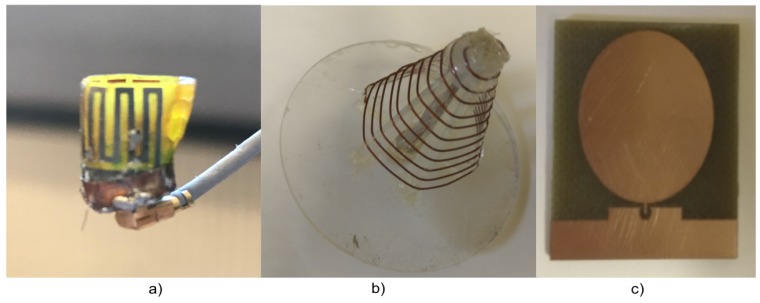
ISM band antennas (**a**) Transmitter (**b**) Eve antenna (**c**) legitimate Receiver.

For ISM band measurements, we used three sets of antennas to perform our measurement campaign. An in-body antenna (transmitter) that replicates the leadless pacemaker transmission, a subcutaneous antenna (legitimate receiver) that is used as a subcutaneous implant, and an external antenna (eve antenna) that replicates an eavesdropper link. Figure 4 shows reflection coefficients of the antennas. The reflection coefficients show good matching among all the antennas around 2 GHz. The antennas used are directional and provided in Figure 5. More details on antennas can be found in [31].

#### 3.1.2. UWB Frequency Band

**Figure 6:**
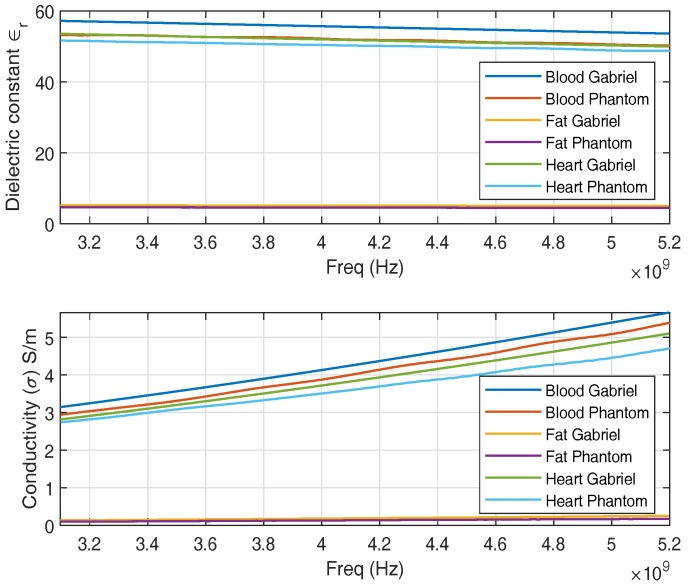
Dielectric Properties of UWB Phantom.

We develop phantoms that mimic the dielectric properties of human heart muscle, blood, and fat in the UWB frequency band. Figure 6, shows the dielectric properties of the replicated phantoms. For the UWB band, the heart phantom is composed of 54.2% acetonitrile and 1.07% salt in water [32]. Similarly, fat and blood phantom is altered to fit for UWB frequency band.

**Figure 7:**
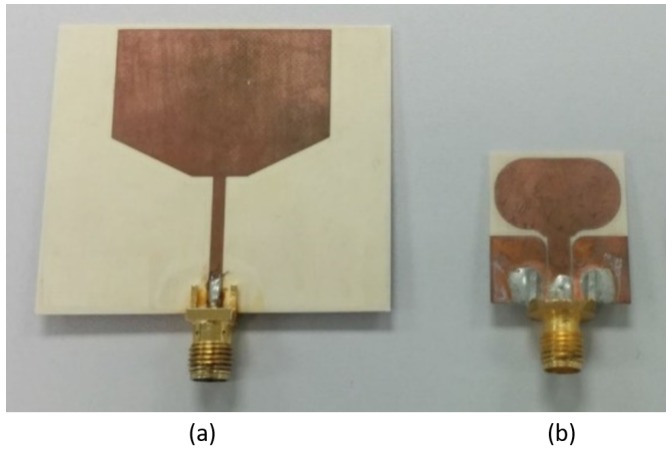
UWB antennas (**a**) Eve Antenna (**b**) Transmitter and Legitimate receiver.

**Figure 8:**
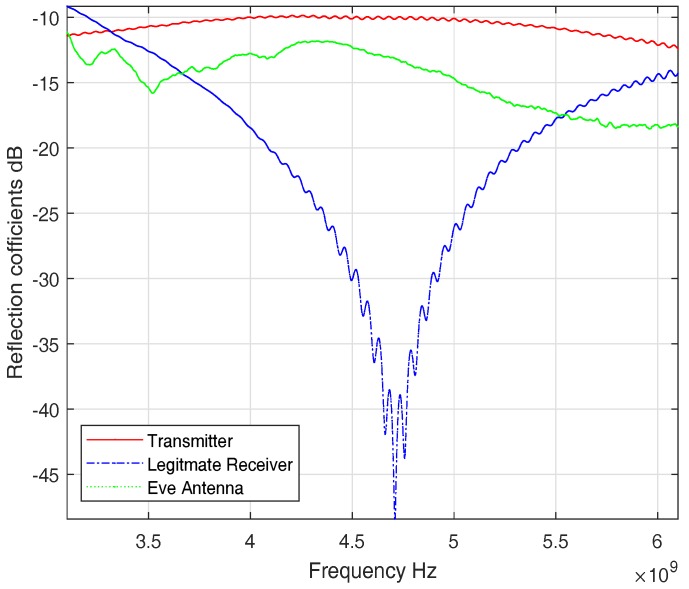
S-parameters of UWB antennas.

Three sets of UWB antennas are used, similar to the ISM band, one implanted (Transmitter), one subcutaneous (legitimate receiver) and one external antenna (eve antenna). Figure 7 shows the antennas used. Figure 7b is the transmitter antenna and a similar antenna is used as the legitimate receiver. The transmitter and legitimate receiver have dimensions of 2.3 × 2 cm${}^{2}$ whereas the eve antenna dimensions are 5 × 4.4 cm${}^{2}$. All the antennas have a quasi-omnidirectional radiation pattern. More details on the antennas are provided in [33,34]. Figure 8 shows the S-parameters of the antennas. All the antennas have transmission parameters of less than −10 dB, which is considered to be a very efficient transmission parameter.

## 4. Measured Channel Models

In this section, using the measurement setup, the IB2IB and IB2OFF channel models are determined for the ISM and UWB frequency bands.

As mentioned earlier, the phantoms prepared are different for different frequency bands. Therefore, while conducting the experiments for ISM and UWB frequency band, phantoms and antennas are replaced. First, we used a single-layer phantom that contains only heart muscle for ISM and perform the IB2IB and IB2OFF measurements. Then we added the fat layer and repeated the measurements. Once the results are analyzed, a small difference in results with and without fat is observed. Considering this fact, for UWB frequency band instead of using only heart muscle, we performed experiments directly with heart muscle and fat layer for IB2IB and IB2OFF measurements and afterwards the blood layer is added (We wanted to use blood for ISM experiments as well, but the antenna was broken while performing that experiment due to which the path loss containing blood phantom for ISM band is not presented).

**Figure 9:**
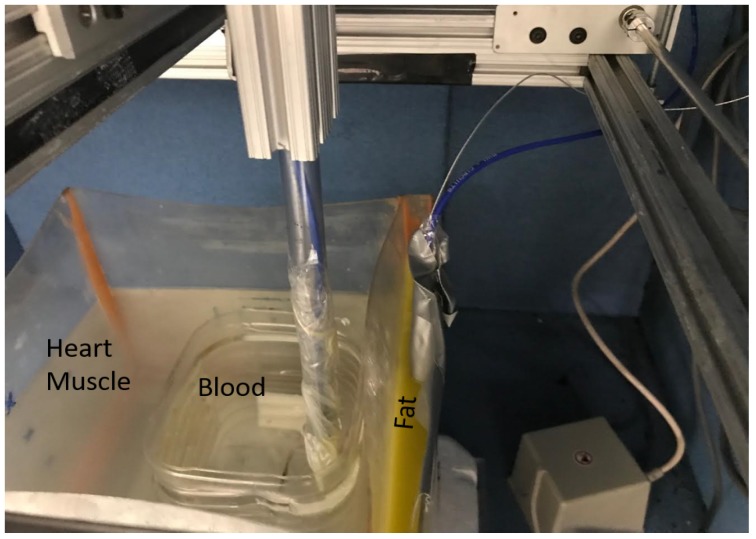
Multilayer phantom container inside anechoic chamber.

**Figure 10:**
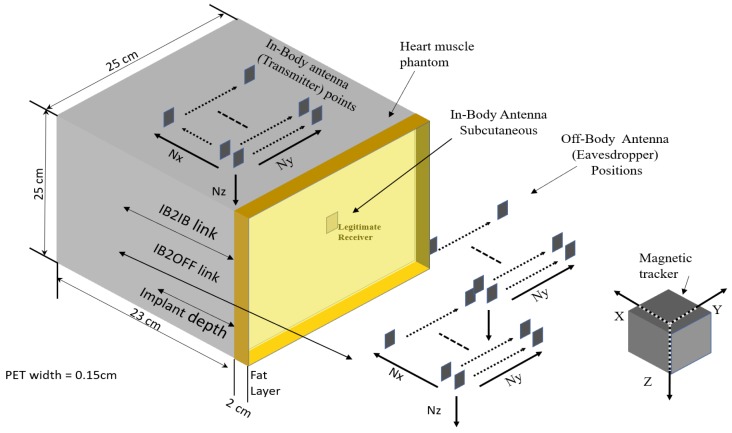
Measurement Grid points.

The measurements for legitimate link (IB2IB) are performed by implanting a transmitter antenna inside the liquid phantom, whereas the subcutaneous antenna or legitimate receiver is mounted on the wall of the container (subcutaneous). In the case of the fat layer, the subcutaneous antenna is placed inside the fat layer as shown in Figure 9. Furthermore, for legitimate link measurements, the implanted antenna (transmitter) is moved in different grid points along the x, y and z axis with a step size of Δx,Δy & Δz with total grid points of (Nx, Ny, Nz) as shown in Figure 10. For eavesdropper link measurement, the implanted antenna (transmitter) is fixed at a certain implant depth and eve antenna is moved across different grid points outside the phantom container to replicate different eavesdropper positions. In addition, for each measuring point, five snapshots are taken and then averaged to enhance the SNR. We would like to highlight the fact that the entire experiment takes around three weeks.

### 4.1. Path-Loss Models

From the measurements, we obtain the channel transfer functions, which help in determining the path loss for single-layer (heart muscle) and multilayer phantoms (fat + heart + blood) in both frequency bands. We measured the forward transmission coefficient $S_{21}$ for N resolution points (see Table 1); the path loss per spatial position can be expressed as,

(1) $$\begin{array}{c} \begin{array}{c} PL_{i}\left(dB\right)={\left|h_{i}\right|}^{2}=10\times \log_{10}\left(\sum_{k=N}\frac{|H{\left(f_{j}\right)}_{k}{|}^{2}}{N}\right),\\ \;i\epsilon (r,e)\\ j\epsilon (ISM,UWB) \end{array} \end{array}$$

$H\left(f_{j}\right)=\left|S_{21}\right|e^{-j∠S_{21}}$, where $|S_{21}|$ and $∠S_{21}$ are module and phase of transmission coefficient. “i” represents legitimate link (r) and eavesdropper link (e). Similarly, “j” represents the frequency band (j = ISM or UWB).

#### 4.1.1. ISM Band

##### 4.1.1.1. IB2IB or Legitimate Link

**Figure 11:**
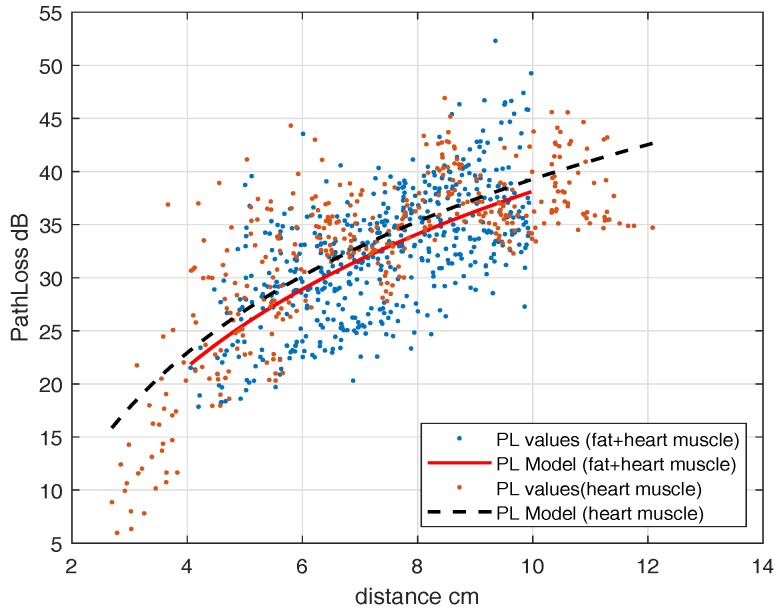
Path-loss legitimate link (IB2IB, ISM).

For the legitimate link, the receiver antenna is mounted on the inner surface of the container’s wall for a single-layer phantom that contains only heart muscle, and the transmitting antenna is moved in different grid points inside the phantom (Figure 10). A leadless pacemaker is considered to be an implanted antenna whereas an antenna fixed on the wall of a container is considered to be a subcutaneous implant. The measured frequency band is 1.7 GHz to 2.5 GHz for the ISM band, but we only take the narrowband part in which the transmitter’s $S_{11}$ is below −6 dB. Thus, only those measurements of $S_{21}$, for which the $S_{11}$ reflection coefficient is below −6 dB, are taken into account. The resulting measured frequency band is 1.946–2.072 GHz as can be seen in Figure 4. The obtained path loss can be modeled as a distance-dependent logarithmic function and can be expressed as

(2) $$\begin{array}{c} \begin{array}{c} PL_{dB}=PL_{d_{0}}+10\times n\times log_{10}\left(\frac{d}{d_{0}}\right)+N\left(\mu ,\sigma \right) \end{array} \end{array}$$

where, $d_{0}$ = 4 cm, $PL_{d_{0}}$ = 22.9284 dB, n=4.12 and $N\left(\mu ,\sigma \right)=\left(-3.42\times 10^{-15},7.3002\right)\approx \left(0,7.3002\right)$. This model is valid for legitimate link distances from 2.7–12 cm. The observed randomness is because of measurements at different angles from the transmitting antenna. For a 2-layer phantom, we fill the portion of the container with a fat layer (Figure 9 except blood layer) and mounted the subcutaneous antenna in the fat layer. We determined the path-loss model, similar to (2). The parameters are $d_{0}$ = 4 cm, $PL_{d_{0}}$ = 21.85 dB, n=4.12 and $N\left(\mu ,\sigma \right)=\left(-4.6\times 10^{-16},4.5\right)\approx \left(0,4.5\right)$. Figure 11 shows the path-loss models both for single-layer and multilayer phantoms together, in which dots are path-loss measurements and the lines are the fitted model. It can be seen that because of the low value of fat dielectric constant, it does not greatly change the path loss model.

##### 4.1.1.2. IB2OFF or Eavesdropper Link

**Figure 12:**
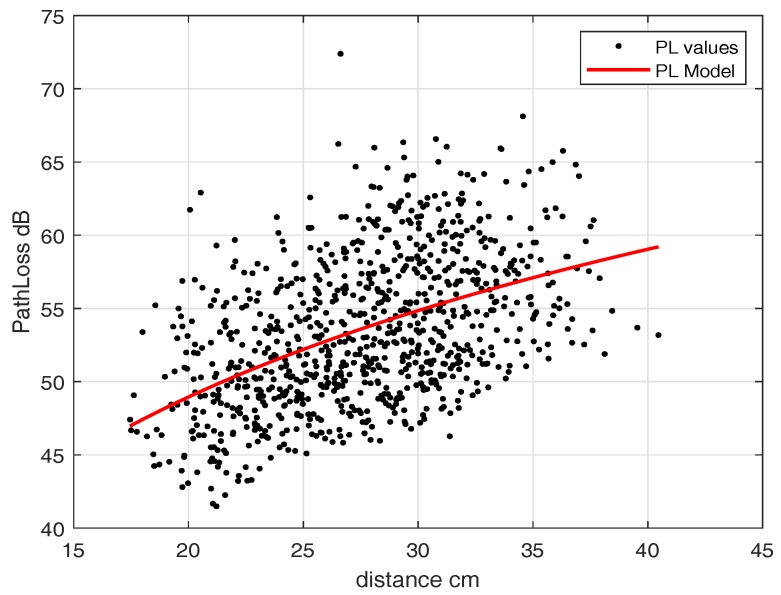
Path-loss eavesdropper link (IB2OFF, ISM).

As we noticed a slight difference in path-loss model with and without fat layer for IB2OFF measurements, we used the single-layer phantom (heart muscle). To find the path loss for the off-body link, we fixed the implanted antenna inside the heart phantom at an implant depth of 11.5 cm and moved the external antenna along the grid points as shown in Figure 10. This replicates the scenario where the leadless pacemaker is implanted at a depth of 11.5 cm inside the body, transmitting to a subcutaneous implant and an eavesdropper outside the body trying to eavesdrop the communication. Similarly to IB2IB, we take $S_{21}$ measurements for narrowband where matching occurs. The path-loss model obtained can be expressed in terms of distance-dependent logarithmic function (2) and the parameters are $d_{0}$ = 17.45 cm, $PL_{d_{0}}$ = 46.97 dB, n=3.352 and $N\left(\mu ,\sigma \right)=\left(-1.17\times 10^{-15},4.40235\right)\approx \left(0,4.40235\right)$. Figure 12 shows the path-loss model for mentioned implant depth. This path-loss model is valid for a distance range of 17.5–40 cm. After 40 cm, a free-space path-loss model can be applied.

#### 4.1.2. UWB Band

#### 4.1.2.1. IB2IB or Legitimate Link

**Figure 13:**
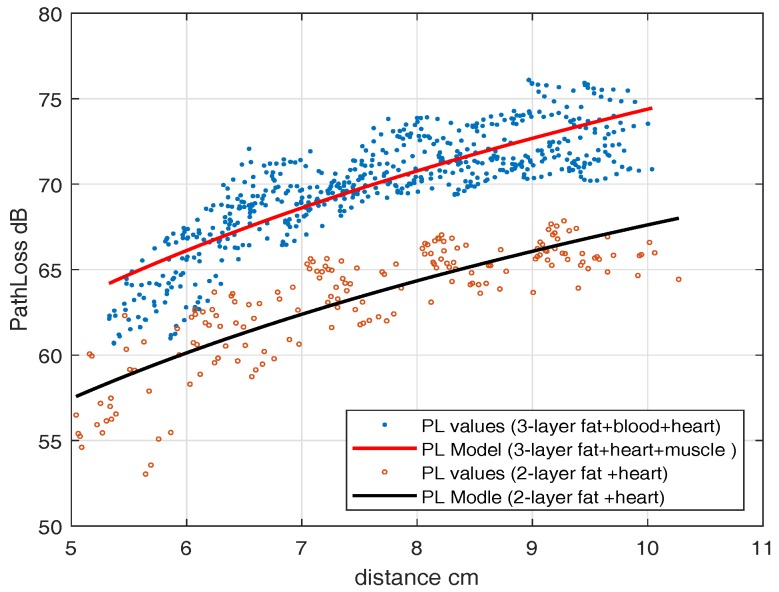
Path-loss legitimate link with and without blood (IB2IB, UWB).

For the UWB frequency band, first, an experiment with a heart muscle and fat layer is performed which is then followed by the blood phantom. To find the IB2IB path-loss model, we fixed an in-body antenna (subcutaneous) in the fat layer and move another in-body antenna to different grid points. Figure 9 shows the placement scenario inside an anechoic chamber. The path-loss model parameters for the IB2IB scenario containing all three phantom layers (heart muscle, blood, and fat) are $d_{0}$ = 4 cm, $PL_{d_{0}}$ = 59.54 dB, n=3.7284 and $N\left(\mu ,\sigma \right)=\left(-1.445\times 10^{-14},1.9675\right)\approx \left(0,1.9675\right)$. This path-loss model is valid for distances of 1 cm–10 cm. Similarly the parameters for 2-layer phantom (heart muscle and fat), $d_{0}$ = 4 cm, $PL_{d_{0}}$ = 54.1830 dB, n=3.37 and $N\left(\mu ,\sigma \right)=\left(-2.8442\times 10^{-16},1.5301\right)\approx \left(0,1.5301\right)$. Figure 13 shows the path-loss model with and without blood. A difference of about 5 dB is observed in experiments with and without blood.

#### 4.1.2.2. IB2OFF or Eavesdropper Link

For IB2OFF, we measured the channel only by considering fat and heart muscle. This is because from an eavesdropping perspective the path loss without blood will be the best-case scenario and worst-case scenario for the leadless capsule. However, if we want to have path loss with blood, at each measuring point 5 dB of loss must be added.

**Figure 14:**
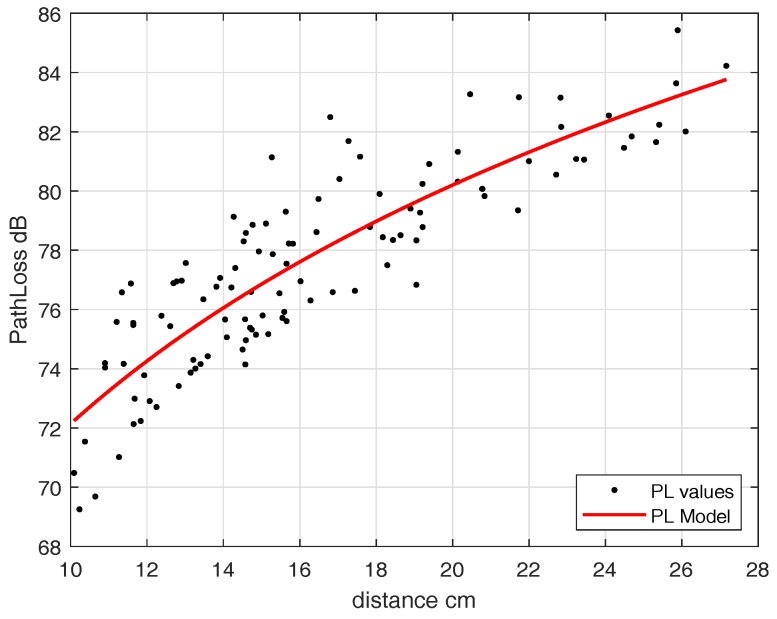
Path-loss Eve link without blood (IB2OFF UWB).

We fixed an in-body antenna at an implant depth of 7 cm and then moved the off-body antenna to different points. Figure 14 shows the path-loss model of the off-body scenario. The parameters in terms of log distance model are $d_{0}$ = 10 cm, $PL_{d_{0}}$ = 72.24 dB, n=2.67 and $N\left(\mu ,\sigma \right)=\left(-1.1627\times 10^{-15},1.6328\right)\approx \left(0,1.6328\right)$. In the case of UWB, the IB2OFF path-loss model is valid for 10–27 cm, whereas beyond 27 cm the free-space path-loss model can be applied.

Table 2 provides summary and comparison of path-loss models for ISM and UWB frequency band.

## 5. Estimating Eavesdropping Risk (Risk Analysis)

This section focuses on estimating eavesdropping risk for next-generation leadless pacemakers in the case of using ISM and UWB band for RF communication. As mentioned earlier, our system consists of an eavesdropper, an IMD and a subcutaneous node as shown in Figure 1. We consider implanted leadless capsules inside the right ventricle of a human heart. This leadless capsule transmits un-encrypted data to the subcutaneous implant, where the eavesdropper tries to eavesdrop the communication. In case of IMDs, Eve can be categorized as

- An eavesdropper, where eavesdropping legitimate transmission can be an active or passive node.
- A possibility of single eavesdropper, or part of a well-organized group.
- An external intruder, but there can be the case where an eavesdropper is from inside the system e.g., physician, hospital administration, equipment manufacturers etc., because they have the benefit of being close to patient.

It can be seen that each eavesdropper may have different intentions or goals. There may be the possibility that one eavesdrops just to get the private information of a patient or there may be a case where there is competition between manufacturers to obtain information on IMD equipment. An eavesdropper may have higher capabilities than that of legitimate nodes such as higher computational power, higher antenna gains, etc. In this work, we consider a single passive eavesdropper with the same capabilities as the legitimate node trying to eavesdrop the communication. We also consider a case where the eavesdropper has higher antenna gain.

### 5.1. Eavesdropper Model

In this section, the probability of successful eavesdropping $\left(P_{se}\right)$ is provided by considering the path-loss models depicted during our measurement campaign, both for UWB and the ISM frequency band. To find $P_{se}$, we use an approach of channel capacity as a measure. Using channel capacity as a basis, for a given information rate (R), there exists a minimum received power to successfully decode the transmission based on a certain threshold SNR. Using this concept, the channel’s capacity is expressed by Shannon capacity formula as

(3) $$\begin{array}{c} C=B\times \log_{2}\left(1+\gamma_{th}\right) \end{array}$$

where *B* is channel bandwidth, *C* is capacity and $\gamma_{th}$ is the threshold SNR. To determine the threshold SNR required to support the information rate (R), (3) can be expressed as

(4) $$\begin{array}{c} \gamma_{th}\left(R\right)>2^{\frac{R}{B}}-1 \end{array}$$

Thus, when SNR at the input of a receiver chain falls below certain threshold level [35], the communication can be termed as in outage and can be expressed as

(5) $$\begin{array}{c} P_{out}\left(\gamma_{th}\right)=P\left[\gamma <\gamma_{th}\right] \end{array}$$

where γ is received SNR. Thus, we can say that when the link is an outage then the eavesdropper will not be able to eavesdrop the communication. Thus, an outage probability can be complemented in terms of probability of successful eavesdropping.

### 5.2. Probability of Successful Eavesdropping ($P_{se}$)

To find the probability of successful eavesdropping, received SNR is required, which can be achieved at a particular distance using the IB2OFF channel model, both for UWB and ISM frequency band Sections 4.1.1.2 and 4.1.2.2. The received SNR can be expressed as,

(6) $$\begin{array}{c} \begin{array}{c} \gamma_{dB}=P_{t_{dBm}}-PL{\left(d\right)}_{dB}-{N_{o}B}_{dBm} \end{array} \end{array}$$

where, $N_{o}B$ is the noise power, P${}_{t}$ is transmit power and PL(d) is the IB2OFF path loss at distance (d) (Eve distance). For a communication between legitimate nodes, the value of SNR (γ) should be greater than threshold, otherwise the communication link will be in outage. However, we want an eavesdropper link to be in outage. Using the concept of outage probability (5), we must take certain cutoff thresholds represented by $\gamma_{th}$. The eavesdropper can eavesdrop communication with certain probability, when the SNR (γ) between the leadless capsule and eavesdropper is greater than the set cutoff threshold $\gamma_{th}$. i.e., $\gamma >\gamma_{th}$. The probability of successful eavesdropping can be expressed as,

(7) $$\begin{array}{c} \begin{array}{c} P_{se}=1-p_{out}\left(\gamma_{th}\right) \end{array} \end{array}$$

As SNR (γ) is log normally distributed (Table 2) with mean $\mu_{\gamma }$ and standard deviation $\sigma_{\gamma }$, we can express (7) by Q-function as

(8) $$\begin{array}{c} \begin{array}{c} P_{se}=1-\left(1-Q\left(\frac{\gamma_{th_{dB}}-\mu_{\gamma_{dB}}}{\sigma_{\gamma_{dB}}}\right)\right) \end{array} \end{array}$$

(9) $$\begin{array}{c} \begin{array}{cc} P_{se} & =Q\left(\frac{\gamma_{th}-\mu_{\gamma }}{\sigma_{\gamma }}\right),\\ P_{se} & =1-\varphi \left(\frac{\gamma_{th}-\mu_{\gamma }}{\sigma_{\gamma }}\right),\\ P_{se} & =1-\frac{1}{2}\left(1+erf\left(\frac{\gamma_{th}-\mu_{\gamma }}{\sigma_{\gamma }\sqrt{2}}\right)\right) \end{array} \end{array}$$

The eavesdropper can increase the probability of successful eavesdropping by using high-gain antennas. An antenna with high gain has a reception from greater distances and has higher SNR. However, nothing comes without cost, and higher gain results from larger dimensions of antennas. Different antennas have different antenna gain and aperture relation but in general it can be expressed as

(10) $$\begin{array}{c} \begin{array}{c} A_{e}=\frac{\lambda^{2}G_{e}}{4\pi } \end{array} \end{array}$$

From the eavesdropper perspective, Eve wants to eavesdrop without noticing. However, with large aperture antennas it cannot happen easily. Therefore, the gain of an antenna cannot be increased above certain limits e.g., if we want to have an antenna gain of 20 dBi then the effective aperture of an antenna will be around 18 cm${}^{2}$ (for frequency of 2 GHz) which cannot go unnoticed within the short distance of a patient.

#### 5.2.1. $P_{se}$ for ISM and UWB Frequency Band

**Figure 15:**
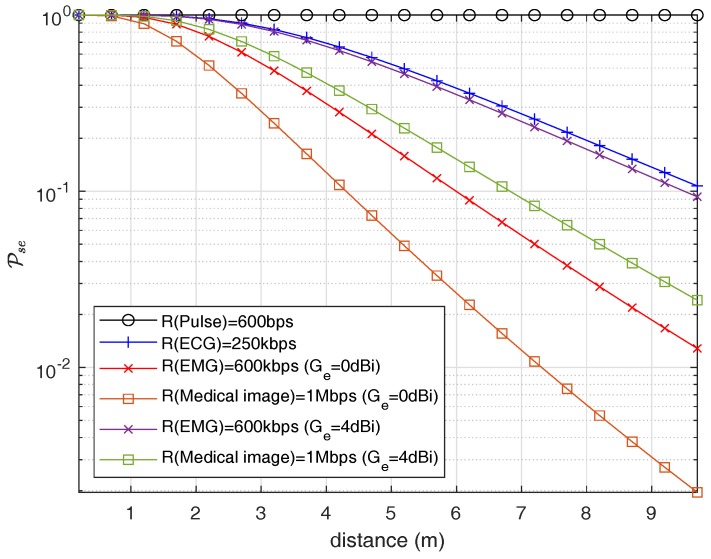
Probability of Successful eavesdropping w.r.t Eve Distance (ISM Band).

To find the eavesdropping risk, path loss at different eavesdropper distances is determined using (1) and the model parameters provided in Table 2 for an eavesdropper link. This helps determining the received SNR for fixed transmitted power. In addition, we also measured the receiver sensitivity for a bandwidth of 1 MHz, which is found to be −90 dBm. Furthermore, different information rates (R) are considered for communication between legitimate nodes, and determine corresponding cutoff thresholds by using (4). We assume the information rates that reflect the real-life application rates such as EMG, ECG, and pulse rate, that are listed in Table 3 [36]. Finally, using (9), we determine the probability of successful eavesdropping risk for a cardiac leadless pacemaker communicating to a subcutaneous implant. Figure 15 shows the probability of successful eavesdropping with varying eavesdropper distance for the ISM frequency band. We consider a channel bandwidth of 1 MHz. The probability of a successful eavesdropping for an information rate of 600 kbps (EMG) is about 97.68% at an eavesdropper distance of 1.3 m and approaches approx 28.13% at an eavesdropper distance of 4.2 m. Similarly, the eavesdropping risk for heart pulse is about 100% at 1.3 m and 4.2 m, whereas for ECG signal the risk is 99.68% at 1.3 m and 65.93% at 4.2 m. When the information rate goes up, the requirement for threshold SNR increases, resulting in lower eavesdropping probability. Similarly, the effect of an eavesdropper antenna is also considered which shows that increase in eavesdropping risk occurs when the eavesdropper uses a high-gain antenna as shown in Figure 15, one without antenna gain and one with antenna gain of 4 dBi for information rate of medical image and electromyogram (EMG).

**Figure 16:**
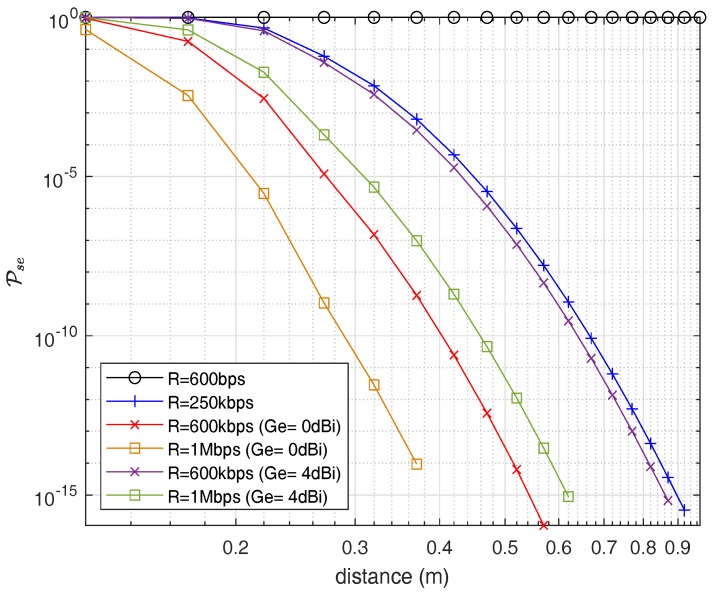
Probability of successful eavesdropping w.r.t Eve distance (UWB).

Furthermore, for the UWB frequency band, Figure 16 shows the probability of successful eavesdropping. Here it would be good to notify that we consider the path-loss model without blood because it would be the best-case scenario for an eavesdropper. Due to high losses in UWB, the eavesdropping risk is considerably less than the ISM band which is one of the advantages of using UWB for in-body networks along with high data rate capabilities. Figure 16 shows the $P_{se}$ on a per-MHz basis similar to ISM frequency band. Similarly, for an information rate of an EMG, the eavesdropping risk drops to 0.2847% at an eavesdropping distance of 0.22 m in the case of the UWB frequency band.

## 6. Secrecy Capacity Analysis

From risk analysis, it is evident to have some secure mechanisms to keep the pacemaker safe from an eavesdropper. As mentioned earlier, this section focuses on the potential of securing pacemakers using a physical-layer security method. Our intention is to use the keyless security method by using the concept of secrecy capacity and secure channel. We deal with exploring the availability of secrecy capacity by using the channel models obtained in Section 4.

Secrecy capacity is the maximum attainable communication rate between legitimate nodes without any leakage of information to the eavesdropper. Consider the wireless system depicted in Figure 1, where a leadless pacemaker communicates with a subcutaneous implant and the eavesdropper attempts to eavesdrop the communication, by recalling [37] for an additive Gaussian wiretap channel, where both channels are corrupted by Gaussian noise in a way that the eavesdropper channel is noisier than legitimate channel i.e., $W_{e}>W_{r}$. Then, the instantaneous secrecy capacity is given as,

(11) $$\begin{array}{c} \begin{array}{c} C_{s}=C_{r}-C_{e} \end{array} \end{array}$$

where,

(12) $$\begin{array}{c} C_{r}=\frac{1}{2}\log_{2}\left(1+\gamma_{r}\right) \end{array}$$

is the instantaneous channel capacity of legitimate link and

(13) $$\begin{array}{c} C_{e}=\frac{1}{2}\log_{2}\left(1+\gamma_{e}\right) \end{array}$$

is the instantaneous channel capacity of eavesdropper link, which follows instantaneous secrecy capacity as,

(14) $$C_{s}=\left\{\begin{array}{cc} {\left[\frac{1}{2}\log_{2}\left(1+\gamma_{r}\right)-\frac{1}{2}\log_{2}\left(1+\gamma_{e}\right)\right]}^{+}, & \mathrm{if}\;\gamma_{r}>\gamma_{e}.\\ 0, & \mathrm{otherwise}. \end{array}\right.$$

$\gamma_{r}$ is legitimate channel (IB2IB) SNR and $\gamma_{e}$ is eavesdropper channel (IB2OFF) SNR. $C_{s}$ is positive when $\gamma_{r}>\gamma_{e}$, which means that the legitimate nodes can communicate securely at that positive secrecy rate. Furthermore, SNR of each link can be expressed as

(15) $$\begin{array}{c} \begin{array}{c} \gamma_{i}=\frac{P_{t}\times {\left|h_{i}\right|}^{2}}{W_{i}},\;i\epsilon \left(r,e\right) \end{array} \end{array}$$

where, $P_{t}$ is transmitted power, $|h_{r}{|}^{2}$, $|h_{e}{|}^{2}$ are channel gains of respective links and W is noise power. As we observed, the channel gains follow log-normal distribution. Thus, $\gamma_{r}$ and $\gamma_{e}$ will also follow the log-normal distribution at any measuring point with mean and standard deviation ($\mu_{r}$,$\sigma_{r}$) and ($\mu_{e}$,$\sigma_{e}$), respectively. The fundamental parameters in the context of secrecy capacity are probability of positive secrecy capacity ($P_{pc_{s}}$) and outage probability of secrecy capacity ($OP_{c_{s}}$). When the legitimate link SNR is better than the eavesdropper link, the secrecy capacity is positive and can be referred to as positive secrecy capacity. The outage probability of secrecy capacity is the probability of outage for certain fixed secrecy rate (Rs) with respect to eavesdropper distance. As $\gamma_{r}$ and $\gamma_{e}$ are mutually independent and log normally distributed, then for single realization of a legitimate channel and eavesdropper channel, the probability of positive secrecy capacity can be expressed as,

(16) $$\begin{array}{c} \begin{array}{cc} P(C_{s}>0) & =P(\gamma_{r}>\gamma_{e}) \end{array} \end{array}$$

To find $OP_{c_{s}}$ and $P_{pc_{s}}$ together, if we consider a certain fixed secrecy rate ($R_{s}$) then the outage probability can be expressed as

(17) $$\begin{array}{c} \begin{array}{cc} P(C_{s}<R_{s}) & =1-P(C_{s}>R_{s}) \end{array} \end{array}$$

Thus, by using definitions of probability,

(18) $$\begin{array}{c} \begin{array}{c} P\left(C_{s}>R_{s}\right)=P\left(\log_{2}\left(\frac{1+\gamma_{r}}{1+\gamma_{e}}\right)>R_{s}\right)\\ =P(\gamma_{r}>e^{R_{s}\ln2}\left(1+\gamma_{e}\right)-1)\\ =\int_{0}^{\infty }f_{\gamma_{e}}\left(\gamma_{e}\right)\left(\int_{e^{Rs\ln2}\left(1+\gamma_{e}\right)-1}^{\infty }f_{\gamma_{r}}\left(\gamma_{r}\right)d_{\gamma_{r}}\right)d_{\gamma_{e}}\\ =\int_{0}^{\infty }f_{\gamma_{e}}\left(\gamma_{e}\right)\left(1-F_{\gamma_{r}}\left(e^{Rs\ln2}\left(1+\gamma_{e}\right)-1\right)\right)d_{\gamma_{e}}\\ =1-\int_{0}^{\infty }f_{\gamma_{e}}\left(\gamma_{e}\right)F_{\gamma_{r}}\left(e^{Rs\ln2}\left(1+\gamma_{e}\right)-1\right)d_{\gamma_{e}}\\ =e^{Rs\ln2}\int_{0}^{\infty }F_{\gamma_{e}}\left(\gamma_{e}\right)f_{\gamma_{r}}\left(e^{Rs\ln2}\left(1+\gamma_{e}\right)-1\right)d_{\gamma_{e}} \end{array} \end{array}$$

where,

(19) $$\begin{array}{c} \begin{array}{c} F_{\gamma_{r}}\left(\gamma_{r}\right)=1-Q\left(\frac{\ln\gamma_{r}-\ln\mu_{\gamma_{r}}}{4a}\right) \end{array} \end{array}$$

(20) $$\begin{array}{c} \begin{array}{c} F_{\gamma_{e}}\left(\gamma_{e}\right)=1-Q\left(\frac{\ln\gamma_{e}-\ln\mu_{\gamma_{e}}}{4b}\right) \end{array} \end{array}$$

$F_{\gamma_{r}}\left(\gamma_{r}\right)$ and $F_{\gamma_{e}}\left(\gamma_{e}\right)$ are cumulative distribution functions of $\gamma_{r}$ and $\gamma_{e}$. In addition, where, $\ln E(\gamma_{e})$ is the mean SNR of eavesdropper link and $\ln E(\gamma_{r})$ is mean SNR for legitimate link (see Equation(15)) and $a=\frac{\sigma_{r}\ln10}{40}$ and $b=\frac{\sigma_{e}\ln10}{40}$, where $\sigma_{e}$ is the channel deviation of eavesdropper link and $\sigma_{r}$ is of legitimate link provided in Table 2. Substituting in (18)

(21) $$\begin{array}{c} \begin{array}{c} P\left(C_{s}>R_{s}\right)=\frac{e^{Rs\ln2}}{4a\sqrt{2\pi }}\int_{0}^{\infty }\frac{1}{e^{Rs\ln2}\left(1+\gamma_{e}\right)-1)}\times \left(1-Q\left(\frac{1}{4b}\ln\frac{\gamma_{e}}{\mu_{\gamma_{e}}}\right)\right)\times \\ \exp\left(\frac{1}{2}{\left(\frac{1}{4a}\ln\left(\frac{e^{Rs\ln2}\left(1+\gamma_{e}\right)-1}{\mu_{\gamma_{r}}}\right)\right)}^{2}\right)d_{\gamma_{e}} \end{array} \end{array}$$

Equation (21) can be evaluated by numerical methods. The preposition obtained is

(22) $$\begin{array}{c} \begin{array}{c} P\left(C_{s}<Rs\right)=Q\left(\frac{\ln\frac{\mu_{\gamma_{r}}}{\mu_{\gamma_{e}}}+8\left(b^{2}-a^{2}\right)-Rs\ln2}{4\sqrt{a^{2}+b^{2}}}\right) \end{array} \end{array}$$

The proof of (22) is provided in [38] and for convenience also in Appendix A. For strictly positive secrecy capacity $R_{s}$ is set to 0, Thus, (22) can be expressed as [38]

(23) $$\begin{array}{c} \begin{array}{c} P\left(C_{s}>0\right)=1-Q\left(\frac{\ln\mu_{\gamma_{e}}-\ln\mu_{\gamma_{r}}+8\left(b^{2}-a^{2}\right)}{4\sqrt{a^{2}+b^{2}}}\right) \end{array} \end{array}$$

Based on legitimate node and eavesdropper location, $\gamma_{r}\alpha \frac{1}{d_{r}^{n}}$ and $\gamma_{e}\alpha \frac{1}{d_{e}^{n}}$. Thus, if $d_{e}>>d_{r}$ and $\gamma_{r}>>\gamma_{e}$, then $P(C_{s}>0)\approx 1$. In our case scenario, the legitimate nodes are inside the body, whereas an eavesdropper is outside the body, due to which probability of secrecy capacity is depicted in near proximity of implanted devices.

### 6.1. Probability of Positive Secrecy Capacity ($P_{pc_{s}}$)

**Figure 17:**
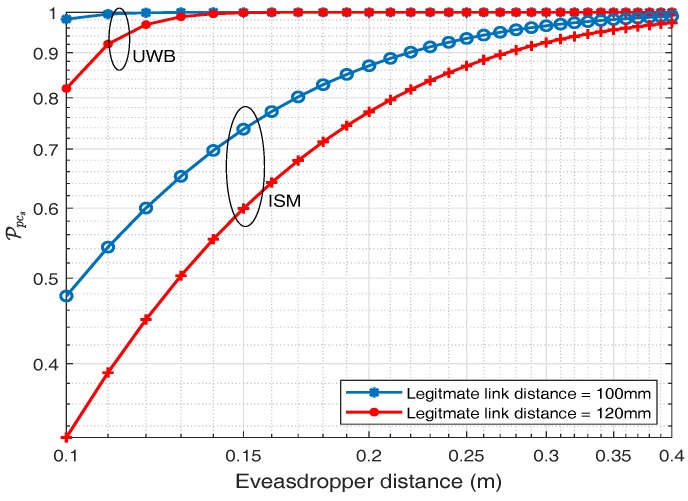
$P_{pc_{s}}$ regarding legitimate link SNR.

The probability of positive secrecy capacity for ISM and UWB frequency band is depicted in this section. By using (23), the probability of positive secrecy capacity for different legitimate distance against eavesdropper distance is plotted in Figure 17 for ISM and UWB frequency band. Two fixed distances for legitimate links are considered and for each distance, the probability of positive secrecy capacity is plotted against varying eavesdropping distance. Figure 17, shows that as the eavesdropping distance increases, probability of positive secrecy capacity approaches to one $P(C_{s}>0)\approx 1$. It also shows that if the eavesdropper is exactly at the same distance to that of the legitimate node i.e., 120 mm, there is still about 44.88% probability of positive secrecy capacity and it approaches approximately 97% at eavesdropping distance of 400 mm for an ISM frequency band whereas for UWB frequency band at the same legitimate distance i.e., 120 mm, $P_{pc_{s}}$ is about 96.84% and similarly approaches to 100% at eavesdropping distance of 400 mm. Thus, UWB frequency band has a higher probability of positive secrecy capacity at the same distance in comparison to the ISM frequency band. This is because of higher attenuation values in the UWB frequency band, which turns out to be a friend and not a foe, for secrecy purposes.

### 6.2. Outage Probability of Secrecy Capacity ($OP_{cs}$)

**Figure 18:**
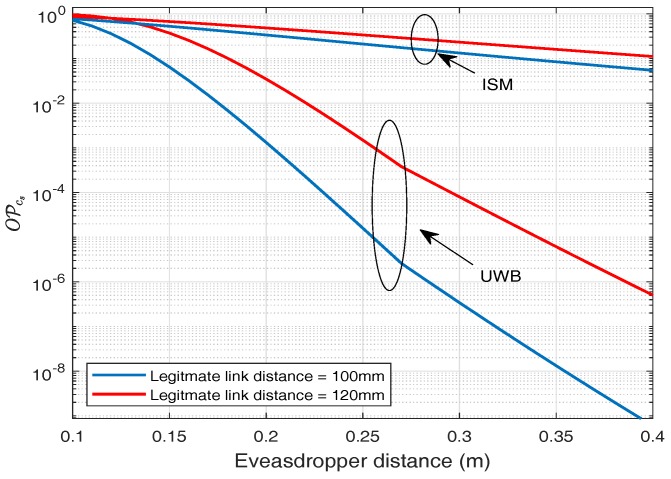
Outage probability of secrecy rate.

Similarly, if we consider certain fixed secrecy rate for our application, then we can determine the outage probability for the given secrecy rate. It is observed that by setting a secrecy rate ($R_{s}$) = 2 bps/Hz and legitimate distance of 120 mm, outage probability is about 80.81% at an eavesdropping distance of 120 mm, whereas at eavesdropping distance of 400 mm it falls to 11.12% for ISM frequency band as shown in Figure 18. For UWB frequency band, considering the same statistics, outage probability at an eavesdropping distance of 120 mm is about 78%, whereas for eavesdropping distance of 400 mm it falls to about $0.5\times 10^{-6}\%$, which shows the rapid decay in outage probability of secrecy rate.

## 7. Discussions

In this section, we discuss the results and compare them for ISM and UWB frequency bands provided as contributions in Section 1. Our findings show that both IB2IB link and IB2OFF link in either case (ISM and UWB) follow the log-normal distribution, which can perfectly represent the real scenario of propagation through different body tissues, e.g., heart muscle, blood, and fat. It is evident that path loss in case of UWB frequency band is higher compared to the ISM band due to which the outage probability of link is also high, resulting in lower cases for eavesdropping. The probability of successful eavesdropping for an information rate of 600 kbps (EMG) is about 97% at an eavesdropping distance of 1.2 m and approaches to approximately 28% at an eavesdropping distance of 4.2 m. For similar rate, the eavesdropping risk drops to 0.2847% at an eavesdropping distance of 0.22 m in case of UWB frequency band.

Similarly, for secrecy capacity analysis where both the links (IB2IB and IB2OFF) play roles, then in the case of UWB the probability of positive secrecy capacity is about 100% at an eavesdropping distance of 200 mm, considering the distance between legitimate nodes to be 120 mm, whereas for a similar scenario, considering ISM band the probability of positive secrecy capacity is about 76%. In addition, if we consider a certain fix secrecy rate Rs = 2 bps/Hz, then for UWB band the outage probability is about 3.4%, whereas for ISM band is about 48.53%, considering the distance between legitimate nodes to be 120 mm.

In fact, UWB has many advantages, including high bandwidth, high data rate, and continuous hopping to make it resilient to interference. In addition, UWB is also considered to be more secure. To eavesdrop in case of UWB is like tracking a person who changes clothes continuously while running at very high speeds. Considering our application of a cardiac leadless pacemaker, very little bandwidth and data rate is required, due to which transmission in an ISM band is more feasible. Furthermore, it is analyzed that the positive secrecy capacity still can be achieved, even when the eavesdropper is as close as 12 to 15 cm from an implanted node. It is been found that even if the eavesdropper is exactly the same distance as an implanted node to which the leadless capsule is transmitting, the probability of positive secrecy capacity is still about 45% and approaches approximately 97% at eavesdropping distance of 40 cm for the ISM band, whereas for UWB the stats are 96% and 100% at 40 cm. With the advent of positive secrecy capacity, Gaussian wiretap codes or LDPC codes can be used to achieve this secrecy rate.

The channel is considered to be secure if the transmissions are done on a secrecy rate. This physical-layer security (PLS) method can also be used along with conventional encryption algorithms on higher layers. If the secure channel rate is low, only encryption keys from higher layers can be shared over available low-rate secure channels, whereas encrypted data communications follows afterwards.

## 8. Conclusions

This work analyzes the eavesdropping risk and the potential of securing next-generation LCPs communicating between implanted nodes via PLS methods. The objective is achieved by adopting the methodology of phantom experiments. In conventional pacemakers, the electrodes in the right atrium and right ventricle are connected via wires to the subcutaneous implant, whereas in the case of a leadless pacemaker the electrodes will transmit wirelessly to the subcutaneous implant, due to which the security is of great concern from an eavesdropper perspective. A three-node model is considered where two nodes are implanted inside the body and an external node located outside the body acts as an eavesdropper.

Human heart-, fat-, and blood-like homogeneous and heterogeneous liquid phantoms are developed to mimic the behavior of electromagnetic wave propagation through the heart. Phantoms developed closely reflect the dielectric properties of heart, fat, and blood in the respective bands. Using these phantoms along with an automated channel measurement mechanism, the channel transfer functions are obtained for a legitimate link and link between implanted node and that of an eavesdropper. Channel measurements are performed for ISM and UWB frequency bands. Furthermore, these channel transfer functions are used to develop path-loss models for both IB2IB link (legitimate link) and IB2OFF link (eavesdropper link) in both bands. Once the path-loss models are depicted, the probability of successful eavesdropping is determined by using the concept of outage probability for different cardiac usable information rates. Afterwards, secrecy capacity analysis is applied to highlight the potential of PLS security methods for wireless cardiac implants.

## Figures and Tables

**Table 1:** Setup parameters.

Band	ISM	UWB
Phantom	Heart muscle, blood & Fat	Heart muscle, blood & Fat
Frequency range	1.7–2.5 GHz	3.1–5.1 GHz
Resolution points	1601	1601
Resolution Frequency	0.5 MHz	1.25 MHz
Intermediate Frequency	3 KHz	3 KHz
Output power	8 dBm	8 dBm
Snapshots per position	Ns = 5	Ns = 5

**Table 2:** Summary of path-loss models.

	ISM Band			UWB Band		
**Parameters**	**Legitimate Link (IB2IB)**	**Legitimate Link (IB2IB)**	**Eve Link (IB2OFF)**	**Legitimate Link (IB2IB)**	**Legitimate Link (IB2IB)**	**Eve Link (IB2OFF)**
Layers	1	2	1	3	2	2
$PL_{d_{0}}$ (dB)	22.92	21.85	46.97	59.54	54.138	72.24
n	4.12	4.12	3.352	3.7284	3.37	2.67
σ	7.3002	4.5	4.4023	1.9675	1.5301	1.6328
μ	0	0	0	0	0	0
$d_{0}$ (cm)	4	4	17.45	4	4	10
Distance Range (cm)	2.7–12	2.7–12	17.5–40	1–10	1–10	10–27

**Table 3:** IMD data traffic.

Sensing Parameter	Required Data Rate
Heart rate	1 sample/s or 600 bps
Medical image	1 Mbps
Blood pressure	1.2 kbps
EMG	600 kbps
EEG	4.2–32 kbps
ECG	250 kbps

## Abbreviations

The following abbreviations are used in this manuscript:

LCP	Leadless Cardiac Pacemaker
IB2IB	In-Body to In-Body
IB2OFF	In-Body to Off-Body
ISM	Industrial Scientific and Medical Frequency Band
UWB	Ultrawide Band
WBAN	Wireless Body Area Network
PLS	Physical-Layer Security
RF	Radio Frequency

## Appendix A

(A1) $$\begin{array}{c} \begin{array}{c} P\left(C_{s}>0\right)=\frac{1}{4a\sqrt{2\pi }}\int_{0}^{\infty }\frac{1}{\gamma_{e}}\times \left(1-Q\left(\frac{1}{4b}\ln\frac{\gamma_{e}}{n}\right)\right)\\ \times \exp\left(\frac{1}{2}{\left(\frac{1}{4a}\ln\left(\frac{\gamma_{e}}{m}\right)\right)}^{2}\right)d_{\gamma_{e}} \end{array} \end{array}$$

Consider,

(A2) $$\begin{array}{c} \begin{array}{c} x=\frac{1}{4b\sqrt{2}}\ln\left(\frac{\gamma_{e}}{n}\right) \end{array} \end{array}$$

Then (A1) becomes

(A3) $$\begin{array}{c} \begin{array}{c} P\left(C_{s}>0\right)=\frac{b}{a\sqrt{\pi }}\left(\alpha -\beta \right) \end{array} \end{array}$$

where,

(A4) $$\begin{array}{c} \begin{array}{cc} \alpha & =\int_{-\infty }^{\infty }exp\left(-{\left(\frac{b}{a}\right)}^{2}{\left(x+\frac{1}{4b\sqrt{2}}\ln\left(\frac{n}{m}\right)\right)}^{2}\right)dx\\ & =\frac{a\sqrt{\pi }}{b} \end{array} \end{array}$$

(A5) $$\begin{array}{c} \begin{array}{cc} \beta & =\int_{-\infty }^{\infty }Q\left(x\sqrt{2}\right)exp\left(-{\left(\frac{b}{a}\right)}^{2}{\left(x+\frac{1}{4b\sqrt{2}}\ln\left(\frac{n}{m}\right)\right)}^{2}\right)dx \end{array} \end{array}$$

Using Middleton’s work ([39], p. 1072), β can be expressed as

(A6) $$\begin{array}{c} \begin{array}{cc} \beta & =\frac{a\sqrt{\pi }}{b}Q\left(\frac{ln(n/m)}{4\sqrt{a^{2}+b^{2}}}\right) \end{array} \end{array}$$

which follows,

(A7) $$\begin{array}{c} \begin{array}{c} P\left(C_{s}<Rs\right)=Q\left(\frac{\ln\frac{\mu_{\gamma_{r}}}{\mu_{\gamma_{e}}}+8\left(b^{2}-a^{2}\right)-Rs\ln2}{4\sqrt{a^{2}+b^{2}}}\right) \end{array} \end{array}$$

and

(A8) $$\begin{array}{c} \begin{array}{c} P\left(C_{s}>0\right)=Q\left(\frac{\ln\mu_{\gamma_{e}}-\ln\mu_{\gamma_{r}}+8\left(b^{2}-a^{2}\right)}{4\sqrt{a^{2}+b^{2}}}\right) \end{array} \end{array}$$
